# Efficiency in Kinesiology: Innovative Approaches in Enhancing Motor Skills for Athletic Performance 2.0

**DOI:** 10.3390/jfmk9030137

**Published:** 2024-08-14

**Authors:** Vincenzo Sorgente, Diego Minciacchi

**Affiliations:** Kinesiology and Motor Control (Ki.Mo.Co.) Laboratory, Department of Experimental and Clinical Medicine, Physiological Sciences Section, University of Florence, 50134 Florence, Italy; diego.minciacchi@unifi.it

## 1. Introduction

The second edition of the Special Issue titled “Efficiency in Kinesiology: Innovative approaches in enhancing motor skills for Athletic Performance” has been effectively concluded, significantly enriching the discourse on “efficiency in kinesiology” by presenting a diverse array of innovative research findings and methodologies aimed at optimizing athletic performance and motor-skill development (https://www.mdpi.com/2411-5142/8/3/111, accessed on 28 July 2024).

Research from the last few decades has established a solid foundation for enhancing athletic performance through various biomotor and technical training and monitoring methodologies [1,2,3,4]. However, the ever-expanding field of sports science incessantly proposes new methodologies and technologies, as well as their applications, aimed at evaluating, improving, and predicting motor performance. These innovations attract attention from academics, practitioners, and the general public alike. Yet, the adoption of new approaches often outpaces the scientific validation process, resulting in a clash between popular methods that may lack robust scientific support and validated techniques which may fail to gain traction in practical settings.

Given the dynamic nature of sports science and the relentless pursuit of improved competitive performance, the development and dissemination of novel scientific approaches is crucial for both trainers and researchers. A comprehensive understanding of the advantages and limitations of these methods is essential to effectively evaluate, predict, and model athletic performance across various levels, from amateur to elite.

The present Special Issue addresses the critical gaps in our knowledge by exploring innovative approaches to enhancing motor skills and athletic performance. A broad palette of experimental experiences cover a wide range of sports including weightlifting, volleyball, sailing, soccer, running, American football, and gymnastics. Each of these sporting activities present unique challenges, requiring specific training and performance evaluation methodologies.

By examining multifaceted approaches, this latest collection aims to provide a deeper understanding on how to optimize athletic training and performance. A total of fifteen manuscripts were accepted for publication and inclusion in this Special Issue.

## 2. Overview of Published Articles

This collection of studies presents a comprehensive examination of several aspects of athletic performance and biomechanics across different sports and activities, highlighting their critical role in enhancing athletic achievements. The main components of the findings in this new issue of Efficiency in Kinesiology are described below.

Female volleyball players showed notable adaptations in their jump mechanics when transitioning from rigid surfaces to sand, leveraging the stretch-shortening cycle for better performance, underscoring the importance of surface-specific training [5]. Additionally, in physically active women, reliability in VO2max measurements was high, although individual variations necessitate personalized assessments, emphasizing the need for individualized training protocols [6]. In the realm of running-related injuries, they were shown to be more prevalent among those with higher running volumes and performance-driven motives, indicating the necessity for tailored injury prevention strategies to ensure athletes’ long-term health and performance sustainability [7]. Moreover, high exercise cardiac loads were found to detrimentally impact running speed and autonomic nervous system recovery in collegiate football players, stressing the significance of balanced training and recovery protocols to maintain optimal performance [8]. Furthermore, the cardiac loads of exercise proved to be a reliable predictor of autonomic nervous system deterioration, aiding in the prevention of overtraining and highlighting the value of monitoring physiological loads [9]. Sport-specific training significantly enhanced jump performance in female volleyball players [10], while fitness tests for linear speed were validated as being reliable for monitoring pubertal soccer players, supporting their use in evaluating and improving athletic performance [11]. In rhythmic and artistic gymnastics, specialized training contributed to a superior range of motion in joints and muscle strength in prepubertal athletes, showcasing the benefits of early targeted training on neuromuscular development [12]. The reactive strength index from drop jumps emerged as a strong predictor of weightlifting performance [13], although unstable load back squats did not significantly improve countermovement jump performance, indicating the need for further research in resistance training methodologies [14]. In addition, self-efficacy was closely linked to squat jump performance, reflecting the importance of psychological factors in athletic achievements and suggesting potential areas for intervention [15]. In sailing, performance variables like velocity and maneuvers made were influenced by wind conditions, with notable gender differences in maneuver frequency, emphasizing the need for context-specific performance analysis [16]. Dynamic stretching and proprioceptive neuromuscular facilitation were both effective in enhancing vertical jump height and sprint performance, highlighting effective warm-up techniques [17]. The “sticking region” in squats revealed biomechanical variations based on sex and repetitions, crucial for optimizing training regimens. Finally, the isometric peak torque and rate of torque development provided valuable insights into the neuromuscular fitness of weightlifters, underscoring their predictive power for performance outcomes and the importance of monitoring these metrics for improved training results [18]. The only systematic review of this Special Issue is dedicated to kinesiophobia, a condition marked by an intense fear of physical movement following an injury, presenting a significant barrier to rehabilitation. Central to understanding this condition is the Tampa Scale of Kinesiophobia, a tool to assess the degree of fear and its impact on daily life. These mental factors not only amplify the fear of movement, but also perpetuate a cycle of inactivity and psychological distress. By addressing these underlying issues, particularly through measures that reduce anxiety, bolster self-confidence, and challenge avoidance behaviors, more effective strategies for managing kinesiophobia and promoting physical recovery can be developed [19].

These findings collectively highlight the intricate interplay of biomechanics, training, psychological factors, and environmental conditions in shaping athletic performance, stressing the significance of a holistic approach to athletic development.

## 3. Conclusions

Grounded in the research reported above, several potential directions for future study can establish a proficient and novel framework for the enhancement of athletic performance. In light of this new editorial, we propose the following directions to continue advancing the field of sports science.

First, the application of emerging technologies, such as wearable sensors and machine learning, is a promising avenue to enhance performance evaluation and prediction models. Wearable sensors can provide real-time data on various performance metrics, while machine learning algorithms can analyze these data to offer insights and predict future performance trends.

Additionally, conducting long-term studies to assess the chronic effects of various training methodologies and their transferability to real-world sports settings is crucial. Longitudinal research can reveal the long-term benefits or drawbacks of specific training regimens, helping to fine-tune practices for sustained athlete development.

Furthermore, developing personalized training regimens based on individual biomechanical, physiological, and psycho-cognitive profiles to optimize performance and reduce injury risk remains essential [20,21,22,23,24]. Personalized programs can adapt to changes in an athlete’s condition over time, ensuring continuous optimization and safety.

Moreover, another key direction is encouraging collaboration between sports scientists, coaches, psychologists, and engineers to create holistic training programs that address all facets of athletic performance. Cross-disciplinary efforts can lead to comprehensive training solutions that incorporate physical conditioning, mental resilience, and advanced technological support.

Finally, it is also vital to investigate the impact of early specialization versus diversified training in young athletes to determine the best practices for long-term athletic development. Understanding the balance between specialized and varied training in youths can guide practices that maximize potential and minimize burnout- or overuse-related injuries.

By addressing these areas, future research can provide valuable insights and practical applications, ultimately enhancing the effectiveness of training programs and improving athletic performance across all levels. In particular, it may be worthwhile to promote umbrella reviews to enhance the breadth and depth of scholarly understanding.

In conclusion, we wish to gratefully acknowledge the essential contributions from all of the authors, reviewers, and editors toward this Special Issue. Given the great success of this Special Issue, we have launched a third edition. We believe that this topic has the potential to propel sports science forward by connecting cutting-edge scientific research with practical on-field training methods and experiences.
